# Diagnosis of rectal cancer by Tissue Resonance Interaction Method

**DOI:** 10.1186/1471-230X-10-45

**Published:** 2010-05-12

**Authors:** Alberto Vannelli, Luigi Battaglia, Elia Poiasina, Ermanno Leo

**Affiliations:** 1 Division of General Surgery B, Fondazione IRCCS Istituto Nazionale Tumori, Milan, Italy

## Abstract

**Background:**

Since population screening has the potential to reduce mortality from rectal cancer (RC), novel methods with improved cost-effectiveness warrant consideration. In a previous pilot study, we found that the rapid, inexpensive and non-invasive electromagnetic detection of RC is a highly specific and sensitive technique. The aim of the present prospective study was to evaluate the prediction accuracy of electromagnetic detection of RC.

**Methods:**

304 eligible subjects were consecutively enrolled in our Institute and subjected to electromagnetic detection followed by colonoscopy and histopathologic analysis of biopsies. A putative RC carrier status was attributed to subjects showing an electromagnetic signal < 50 units (U).

**Results:**

RC patients showed a significantly lower electromagnetic signal (40.9 ± 0.9 U; mean ± S.E.) than did non-RC subjects (79.2 ± 1.4 U; P < 2.2e-16). At a threshold < 50 U, electromagnetic detection identified 103 putative patients, whereas colonoscopy detected 108 patients, with an overlap of 91 patients between the two methods. The 15.7% false-negative rate by electromagnetic detection was brought to zero by raising the threshold value to 70 U; on the other hand, such a threshold increased the false-positive rate to 30%.

**Conclusion:**

Electromagnetic detection of RC at a signal threshold < 70 U appears to eliminate false-negative results. Although colonoscopy would still be required in examining the false-positives associated with the < 70 U electromagnetic threshold, the need for this method would be reduced. Thus, electromagnetic detection represents a new accurate, rapid, simple, and inexpensive tool for early detection of RC that merits testing in large population-based programs.

## Background

Population screening programs for the early diagnosis of rectal cancer (RC) have the potential to reduce the incidence and mortality from this disease. Most of these programs are based on stool tests or structural exams [1-3]. The main purpose of the screening should be to detect 90% of the sporadic cases of RC. In a health care system with unlimited financial resources the choice of the type of screening and the suitable population for this examination does not represent a problem. Everywhere, even though there are different health care systems, financial resources are limited and the rectal screening with the current methods could be applied only to a selected population.

On the other hand, the majority of adults are not receiving regular age- and risk-appropriate screenings or have never been screened at all [4]. Despite the fact that the primary barriers to screening are lack of health insurance, lack of physician recommendation, and lack of awareness of the importance of RC screening, the historical evidence shows that adults have different preferences and patterns of use among the available RC screening tests [5,6].

Thus, a less expensive, faster, and less invasive RC screening procedure with a similar or better efficacy, as compared to available methods, would provide a significant advantage for RC prevention in the general population. We recently carried out a pilot study for the identification of RC by electromagnetic detection, a method that is rapid, non-invasive, and inexpensive. As compared to the results of colonoscopy, electromagnetic detection of rectal cancer was highly specific (85%) and highly sensitive (94%) [7]. Herein, by a prospective study we evaluated the prediction accuracy of RC by electromagnetic detection.

## Methods

### Subjects

442 patients have been admitted to our outpatient's Department from January to August 2008 because of gastrointestinal disease or clinical symptoms related to colorectal risk. Exclusion criteria consisted of age younger than 18 years, history of psychiatric illness, and preoperative radiotherapy: 27 patients. Under written informed consent, 415 subjects were recruited consecutively (10 patients refused the protocol). All subjects underwent electromagnetic detection of RC, followed by colonoscopy, The patients completed the examination with computed tomography (positive colonoscopy) or abdominal sonography (negative colonoscopy). The device lets the examination limited to the pelvis and we regarded the rectum cutoff within 15 cm from the anal verge. Biopsy of suspected neoplastic lesions and histopathological exam of the eventual lesions were performed (209 patients), showing that 108 patients carried a rectal cancer whereas 101 patients carried a cancer in the upper gastrointestinal tract (right or left colon); these latter patients were excluded from this study (Table 1). The study protocol was approved by the Institutional Review Board and Ethics Committee of the Foundation IRCCS Istituto Nazionale Tumori. The ClinicalTtrials.gov ID of the study is: NCT00963794.

**Table 1:** Characteristics of controls and rectal adenocarcinoma patients subjected to electromagnetic and colonoscopy detections of rectal cancer^a^

Subject characteristics	Controls	Cases
No. of subjects	196	108
Median age (range)^b^	65 (24-84)	65 (22-85)
Gender		
*Male*	114	66
*Female*	81	42
Diameter of neoplastic lesion (mm)	NA^c^	48.7 ± 1.7^d^
Distance from anal verge (cm)		
*2 - 6*	NA^c^	47
*7 - 10*	NA^c^	33
*11 - 15*	NA^c^	27
Nodal status		
*N0*	NA^c^	59
*N *≥ 1	NA^c^	48

^a ^Definition of RC cases and healthy controls based on the results of the colonoscopy; such exam has been carried out subsequently to the electromagnetic assay of rectal cancer. ^b ^Age in years. ^c ^NA, not applicable. ^d ^Mean ± SE

### Electromagnetic detection of rectal cancer

RC screening was carried out using a Tissue Resonance Interaction Method (TRIMprobe) electromagnetic detector (Galileo Avionica, Turin, Italy), which consists of a nonlinear oscillator placed in a cylindrical probe about 30 cm long, a radiofrequency spectrum analyzer, and dedicated computer software. Detection of RC is based on the decrease of the electromagnetic signal compared to the mean signal obtained in healthy subjects. The test was performed while the patient stood 120 cm from the receiver. The operator was on the opposite side of the examined pelvis. No metallic objects were allowed on the patient and no electronic devices were admitted in the test area. The detector was kept at close contact to the pelvis surface and was moved through six planes, to obtain a scan of the whole pelvis volume. Based on our previous study, we used the electromagnetic detection system at 465 MHz frequency, in a scale from 0 to 255 arbitrary U [7]. Measurement of the electromagnetic signal was carried out in blind and before the colonoscopy analysis.

### Statistical analysis

Quantitative differences in electromagnetic score between RC patients and non-RC subjects were analyzed by the Kruskal-Wallis test. Correlations of the electromagnetic signal with size of neoplastic lesions or their distance from anal verge were expressed as Spearman's rho coefficient. Association between disease status and electromagnetic signal scores was assessed using the Fisher's exact test. Receiver operating characteristic (ROC) curves were generated with ROCR package in R Gui [8].

## Results

This study of 442 subjects enrolled at our Institute due to signs of RC risk was carried out using a blind and a prospective design, with patients undergoing electromagnetic detection followed by colonoscopy. Histopathologic analysis of biopsies revealed that all RC cases were of the adenocarcinoma histotype. Data from 196 patients with negative colonoscopy results and 108 patients with rectal cancer by colonoscopy were available for analysis. The median patient age was 65 (range, 24-84) years for the negative colonoscopy group and 65 (range, 22-85) years for the positive colonoscopy group. All patients with a RC diagnosis have been subjected to computed tomography, which revealed 9 liver metastasis and no other primitive cancer types. All patients with positive colonoscopy were admitted to the hospital with a diagnosis of rectal adenocarcinoma and submitted to surgery.

Patients not carrying a RC, with the exception of 13 subjects, have been subjected to abdominal sonography, which revealed no cancer pathology. However, 10 patients revealed active phlogistic processes: 6 inflammatory bowel disease, 1 anal abscess and 3 fistulas. Since PSA levels were not measured as a screening for prostate cancer, this may be a possible limitation to the study results.

Based on previous findings, we attributed a putative RC carrier status to patients whose electromagnetic score at 465 MHz frequency was below 50 U, which identified 103 putative patients in our cohort (Table 2). Subsequent colonoscopy to define RC patients (cases) and non-RC subjects (controls) detected 108 patients (Table 1); the overlap with electromagnetic detection was of 91 patients (Table 2).

**Table 2:** Association between electromagnetic score settled out at different thresholds and the RC disease status defined by colonoscopy

Electromagnetic signal score	Number of subjects with		*P*^b^
		
	Non-RC^a^	RC^a^	
≥50	184	17	
< 50	12	91	< 2.2e-16
			
≥70	134	0	
< 70	62	108	< 2.2e-16

^a ^By colonoscopy analysis. ^b ^Fisher's exact test.

**Figure 1:**
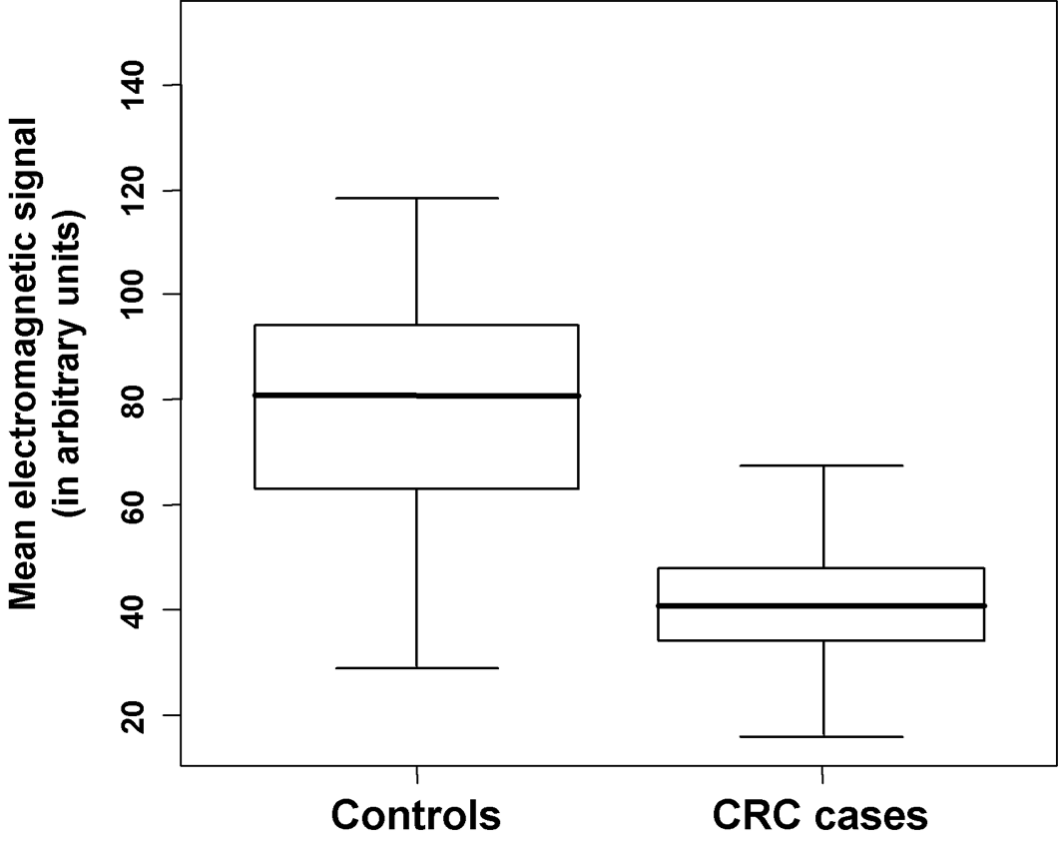
**Lower electromagnetic signal associated with rectal cancer carrier status**. Mean values of the electromagnetic signal (465 MHz frequency), in arbitrary units, were statistically significant lower in RC cases than controls. Case and control categories were based on subsequent colonoscopy and histopathologic exam of biopsies. * P < 2.2e-16, Kruskal-Wallis test.

Mean age and gender distribution were similar between cases and controls (Table 1). RC patients classified by colonoscopy showed a significantly lower electromagnetic signal than did non-RC subjects, i.e., 40.9 ± 0.9 U (mean ± S.E.) versus 79.2 ± 1.4 U (p < 2.2e-16, Kruskal-Wallis test; Fig. 1).

**Figure 2:**
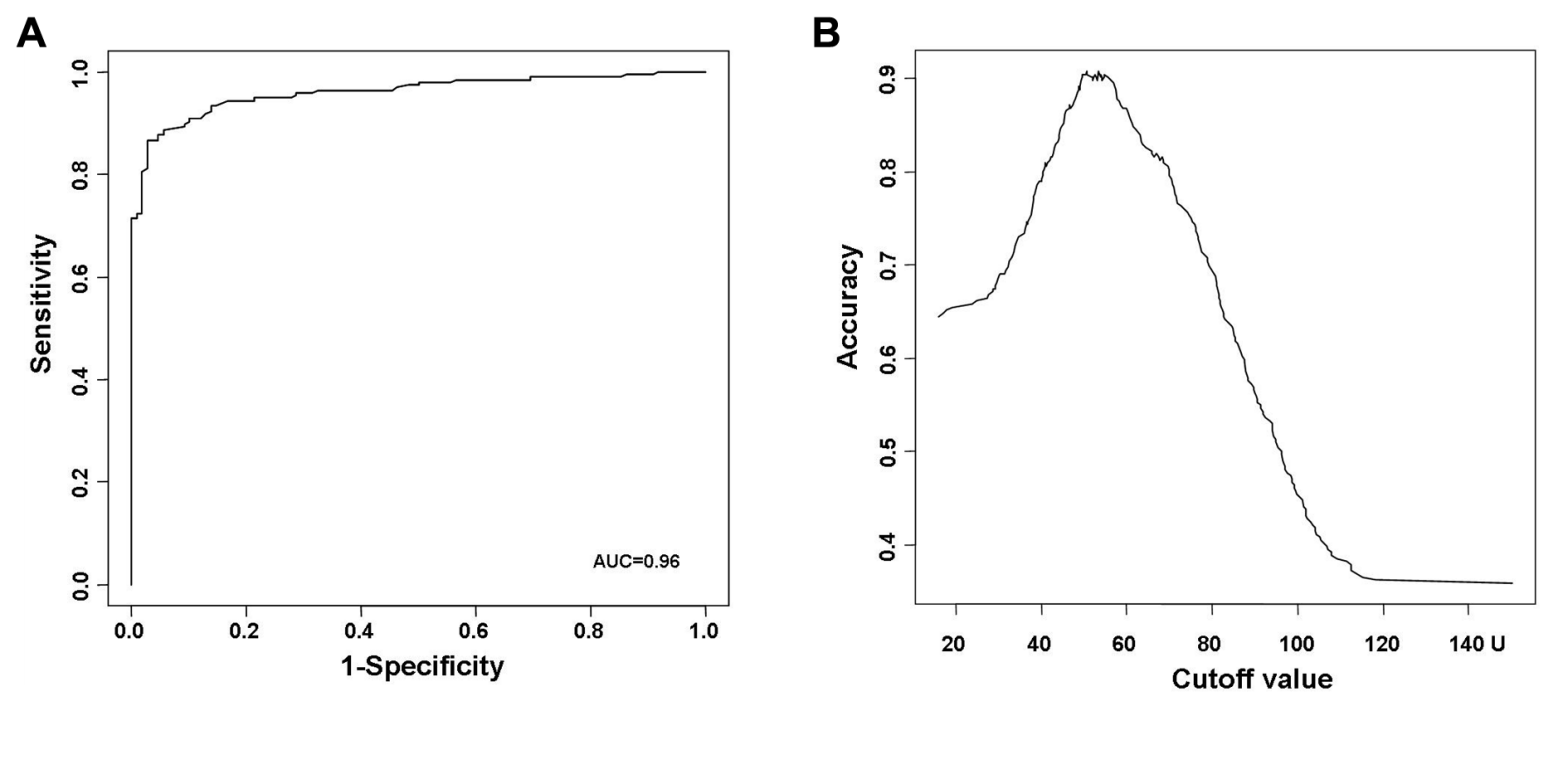
**Visualizations of TRIMprobe electromagnetic signal performance in detecting RC cancer**. (A) ROC curve illustrating the high diagnostic ability of TRIMprobe electromagnetic signal in the differentiation of RC patients versus non-cancer subjects, AUC = 0.96 (95% CI 0.94 - 0.98; P < 2.2e-16). (B) Accuracy of the TRIMprobe electromagnetic signal in the differentiation of RC patients versus non-cancer subjects by the signal cutoff value. The best accuracy is obtained using cutoff values of ~50-55 U.

To evaluate the applicability of TRIMprobe electromagnetic signal as a marker for distinguishing between RC and non-RC disease groups, we performed ROC (Receiver Operating Characteristic) curve analysis. ROC curve showed the diagnostic ability of TRIMprobe electromagnetic signal in the differentiation of RC patients versus non-cancer subjects (AUC = 0.96, 95% confidence interval (CI) 0.94 - 0.98; P < 2.2e-16; Fig. 2A). In our cohort, the sensitivity of the TRIMprobe device for RC was 0.94, specificity was 0.84, negative predictive value was 0.88, positive predictive value was 0.92, and accuracy was 0.90 for the electromagnetic signal cutoff value of 50 U. Indeed, an electromagnetic signal < 50 U was significantly associated with detection of RC by colonoscopy (p < 2.2e-16, Table 2). Analysis of accuracy by cutoff value indicated that ~50-55 U represent the best cutoff values for detection of RC (Fig. 2B)

Since a major goal in screening tests is the minimization of false-negative rates, we identified an electromagnetic threshold, i.e., < 70 U, at which no RC was missed (Table 2). However, at this threshold, 62 (31.6%) of the non-RC subjects were false-positive (Table 2), whose disease or healthy status would have required clarification by colonoscopy.

No association between nodal involvement (N0 versus N ≥ 1) and the value of the electromagnetic signal was observed. A significant inverse correlation was observed between the size of the neoplastic lesions and the value of the electromagnetic signal (Spearman's rho = -0.290, P = 0.002), whereas a significant positive correlation was found between increasing distance from anal verge and the value of the electromagnetic signal (Spearman's rho = 0.362, P = 0.0001).

## Discussion

Since up to 10% of the general population might carry a RC, depending on the age of the population undergoing screening [2,9], new easy and non-expensive methods for population screening for RC may be helpful for early detection of such disease.

The most frequently used screening methods for RC include two general categories: stool tests (tests for occult blood or exfoliated DNA) and structural exams [endoscopy, double-contrast barium enema and computed tomographic colonography (CTC)]. The popular occult blood test is characterized by simplicity, non-invasiveness, and demonstrated mortality benefit but suffers from poor sensitivity, low population compliance, and high costs of follow-up for false-positives. Indeed, in a large study of asymptomatic patients who underwent occult blood testing followed by endoscopy, the sensitivity of the occult blood test for identifying advanced neoplasia was only 24% [10]. Compared to the occult blood test, CTC is much more expensive, whereas this technique has some clear advantages when compared to endoscopy since it is non-invasive, less time-intensive and is associated with a lower risk of complications. However, CTC requires the use of ionizing radiations, a high level of expertise in reading, and has shown wide variations in sensitivity in the various clinical trials [11].

Endoscopy is an invasive, lengthy and expensive procedure requiring adequate clinical infrastructure and medical expertise, and is not without complications. Thus, it represents even a relatively "poor screening" method for RC at the general population level, especially as compared with screening methods, such as the PAP test, for other types of cancer. The ageing of the general population in the Western world, with the consequent increase of people at risk of RC, further makes large screening programs based on colonoscopy unfeasible. Still, early detection of RC can save lives [1,12] and can also decrease the cost of the patient's clinical management, since patients with early neoplastic lesions require simpler surgical resections and treatments than those with advanced disease.

Although endoscopy is generally safe, it is still an invasive procedure with several-fold higher rates of serious complications than for any other commonly used cancer screening test [13]. Repeated examinations over time may incur a substantial cumulative rate of complications [14]. In addition, a relatively small risk (2 to 6%) of RC remains 6 to 36 months after negative colonoscopy, especially when internists and family practice physicians rather than gastroenterologists perform endoscopies [6].

However, in the near term, even greater incidence and mortality reductions could be achieved if a greater proportion of adults received regular screening [13]. Although prospective randomized trials and observational studies have demonstrated mortality reductions associated with early detection of invasive disease, as well as removal of adenomatous polyps, a majority of adults are not receiving regular age and risk-appropriate screening or have never been screened at all [15].

Recent interest has focused on use of TRIMprobe for diagnosis of disease as new screening strategy. This technique is characterized by simplicity, efficacy, and good patient compliance.

In the present prospective study, patients with RC diagnosed by colonoscopy and histopathologic analysis showed significantly lower values of the electromagnetic signal as compared to non-RC patients (Fig. 1). At a signal threshold of 50 U, defined by our previous study as the optimal threshold in discriminating RC from non-RC patients [7], the electromagnetic detection showed a highly significant association with the RC status (Table 2, Figs. 1, 2), thus confirming in an independent cohort our previous findings.

This technology has also been investigated on other cancers, in particular prostate cancers with favorable outcomes [16,17].

The observed inverse correlation between the size of the neoplastic lesions and the value of the electromagnetic signal is consistent with the association between low electromagnetic signal values and high probability of RC, and raises the possibility that RC size represents a factor affecting the sensitivity of RC electromagnetic detection. The positive correlation observed between increasing distance from anal verge and the value of the electromagnetic signal may reflect a decreasing detection power of the device with distance of the lesion or, alternatively, with interference of anatomical structures in the anal region. Further studies are needed to clarify the existence of a dimensional threshold or of a minimal distance from anal verge of RC to be detected by electromagnetic signal.

Notwithstanding the highly significant association between electromagnetic detection and RC status observed using the 50 U signal threshold, the frequency of false-negative results at this threshold was relatively high (15.7%, Fig. 2) and, although much less than the frequency of missing RCs by the fecal occult blood test, too high for population-based RC screening [6,18]. By increasing the signal threshold value to 70 U, we can avoid all false-negative findings in our cohort, thus we can correctly identified all RC cases but increased the frequency of false-positives to about 30% of the non-RC subjects. Thus, follow-up colonoscopy in real- and false-positive subjects would be necessary to characterize the subject's disease status. We are aware of the limitations of our study, since the relatively small size of our series and the consequent low detection power. Also, TRIMprobe was never tested in a multicentric study for the detection of RC and control subjects from general population, without any gastrointestinal symptoms related to RC risk, have not been tested. Other possible limitations that have not been addressed in the present study include operator dependence and the effects of other gastrointestinal diseases.

## Conclusions

Our present findings point to the promise of electromagnetic detection as a simple, accurate, and inexpensive tool for early detection of RC in cancer prevention programs at the general population level. However, the present results represent only a first step and studies in large cohorts and in different populations are needed to further compare the usefulness of this method with other RC screening methods, especially colonoscopy.

In addition, the description of benefits is complicated by different performance characteristics of the variants tests. Moreover, test performances in research settings and in clinical practice may vary. Therefore, we can image in the future the possibility to support the common screening tests with electromagnetic detection.

## Competing interests

The authors declare that they have no competing interests.

## Authors' contributions

AV has full access to all of the data in the study and takes full responsibility for the integrity of the data and for the accuracy of the data analysis. AV and EL conceived of and designed the study and drafted the manuscript. LB and EP were responsible for data acquisition and critically revised the manuscript. EL obtained funding. All authors have read and approved the final manuscript.

## Pre-publication history

The pre-publication history for this paper can be accessed here:

http://www.biomedcentral.com/1471-230X/10/45/prepub
